# Usefulness of Longitudinal Strain to Assess Cancer Therapy-Related Cardiac Dysfunction and Immune Checkpoint Inhibitor-Induced Myocarditis

**DOI:** 10.3390/ph16091297

**Published:** 2023-09-14

**Authors:** Yudai Tamura, Yuichi Tamura

**Affiliations:** 1 Cardiovascular Center, International University of Health and Welfare Mita Hospital, Tokyo 108-8329, Japan; 20m3008@g.iuhw.ac.jp; 2 Department of Cardiology, International University of Health and Welfare School of Medicine, Narita 286-8686, Japan

**Keywords:** longitudinal strain, global longitudinal strain, echocardiography, immune checkpoint inhibitor, immune-related adverse event, myocarditis, cancer therapy-related cardiac dysfunction

## Abstract

Longitudinal strain (LS) measured by echocardiography has been reported to be useful not only for the diagnosis and risk stratification of various cardiac diseases, but also in cardio-oncology. Most previous studies have been conducted on patients undergoing treatment with anthracyclines and human epidermal growth factor receptor 2-targeted therapies. Existing guidelines recommend that global LS (GLS) should be measured before and after the administration of cancer drugs. This recommendation is based on many reports showing that a decline in GLS is indicative of early or mild cancer therapy-related cardiac dysfunction. The main purpose of this article is to provide insight into the importance of LS in patients undergoing cancer treatment and highlight the role of LS evaluation in patients undergoing immune checkpoint inhibitor (ICI) treatment, which is being used with increasing frequency. Among cancer drug therapies, immune checkpoint inhibitors (ICIs) have an important place in cancer treatment and are used for the treatment of many types of cancer. Although the efficacy of ICIs in cancer treatment has been reported, immune-related adverse events (irAEs) have also been reported. Among these irAEs, cardiovascular complications, although rare, are recognized as important adverse events that may result in ICI treatment discontinuation. Myocarditis is one severe adverse event associated with ICIs, and it is important to standardize diagnostic and therapeutic approaches to it. Several studies have reported a relationship between LS and cardiac complications associated with ICIs which may contribute to the early diagnosis of ICI-induced cardiac complications.

## 1. Introduction

The field of cardio-oncology has received substantial attention in recent years, and cancer therapy-related cardiac dysfunction (CTRCD) has become a major clinical concern. Although advances in cancer treatment have improved the prognosis of patients with flat cancer, the incidence of cardiovascular complications is increasing. Among these cardiovascular complications, CTRCD and immune checkpoint inhibitor (ICI)-induced myocarditis are some of the most important clinical implications.

CTRCD can occur in patients treated with potentially cardiotoxic cancer therapies [1,2,3]. CTRCD can lead to heart failure (HF), and the prognosis of patients with HF caused by cancer therapies is poor [4]. The risk of developing CTRCD is lifelong in both adults and children [4]. Moreover, the incidence of CTRCD will continue to increase as the survival time of patients with cancer is prolonged, the incidence of chronic underlying conditions related to cardiovascular disease increases, and novel cancer therapies are developed [5]. Several studies have reported the usefulness of global longitudinal strain (GLS) as a diagnosis of early and mild CTRCD. The usefulness of regional longitudinal strain (LS) as well as GLS has also been reported. Regional LS is calculated from the LS in each of the three layers (basal, mid, and apical) of the left ventricle, and GLS is measured from the LS in the entire left ventricle. Regional LS has the potential to detect unique myocardial damage earlier than GLS. On the other hand, regional LS is evaluated over a narrower area than GLS and must be sensitive to image quality. Since both GLS and regional LS can be measured in one measurement, the time to perform echocardiography is not prolonged.

ICIs are among the drugs that have improved cancer treatment outcomes in recent years, and their use and indications are continuously expanding [6,7]. However, ICI therapy can cause immune-related adverse events (irAEs), of which cardiac irAEs are of particular importance. Myocarditis is one example of a cardiac irAE caused by ICI therapy. Although myocarditis has a low incidence rate in this context, it has a high mortality rate of 27–50% [8,9,10]. Notably, the relationship between cardiac complications, such as myocarditis, and LS in patients undergoing ICI therapy is beginning to be reported.

The main goals of this article are to describe the usefulness of LS assessment in CTRCD; describe irAEs in patients undergoing ICI therapy; and investigate the utility of LS in patients undergoing ICI therapy. We also aim to improve the understanding of the importance of LS in patients undergoing cancer treatment and provide expert insight into the position of LS assessment in patients undergoing ICI therapy, which is expected to receive great research interest in the future.

## 2. CTRCD

A previous study reported that the incidence of CTRCD is approximately 27% with trastuzumab, 11% with sunitinib, and 2% with imatinib, lapatinib, trametinib, and bevacizumab [1]. Anthracyclines are associated with a CTRCD incidence of 2–48% [11,12,13,14,15]. With anthracyclines, 98% of CTRCD cases occur within the first year of treatment [16]. However, simply waiting for a decrease in left ventricular ejection fraction (LVEF) or HF development is clearly not the best approach to CTRCD management; instead, patients should undergo regular echocardiographic follow-up. Current recommendations [17,18] describe the recommended duration of echocardiographic follow-up after treatment with cancer therapies to monitor CTRCD development. LVEF is a well-established echocardiographic measure for the early recognition of cardiotoxic side effects, and it can be used to prevent irreversible CTRCD and HF development. The diagnosis of CTRCD based on LVEF is defined as a reduction in LVEF to below the normal range (Table 1).

LVEF of 50–55% after anthracycline therapy was reported to be associated with a high rate of symptomatic heart failure and cardiac events thereafter (5.6–12.5%) [19,20]. LVEF may, however, be ineffective for the detection of subclinical myocardial dysfunction that later evolves into symptomatic HF [21], as reviewed previously [22]. Among patients with cancer, as well as cancer survivors, the development of cardiac dysfunction (which can present both acutely and chronically) is an important cause of morbidity and mortality. Therefore, more accurate strategies for the early detection of CTRCD are paramount to improve prognosis and patient outcomes.

LS is used in daily clinical practice [17,18]. Myocardial strain reflects the magnitude of the deformation of a defined length of the myocardium during each cardiac cycle relative to its original length [23]. In cardio-oncology, a number of studies have used GLS to detect CTRCD, and its reliability was reviewed in a recent meta-analysis [24], which showed sensitivity and specificity values ranging from 80% to 90%. In this meta-analysis, the median sensitivity of CTRCD detection was 86% with a specificity of 79%, although a study of breast cancer patients reported a sensitivity of 100% and specificity of 93% [25]. Importantly, GLS is indicated as a useful measure for the detection of subclinical CTRCD [26]. A retrospective study reported that GLS-guided cardioprotective drug intervention significantly reduced the incidence of trastuzumab discontinuation due to cardiotoxicity [27]. Additionally, there have been several reports of GLS improvement with the initiation of cardioprotective drugs based on GLS changes during treatment with anthracycline or trastuzumab [28,29]. However, although previous reviews have described the utility of LS as a prognostic measure in patients undergoing cancer therapy, they have tended to focus on older cancer therapies, such as anthracyclines and trastuzumab [23].

## 3. LS

**Figure 1:**
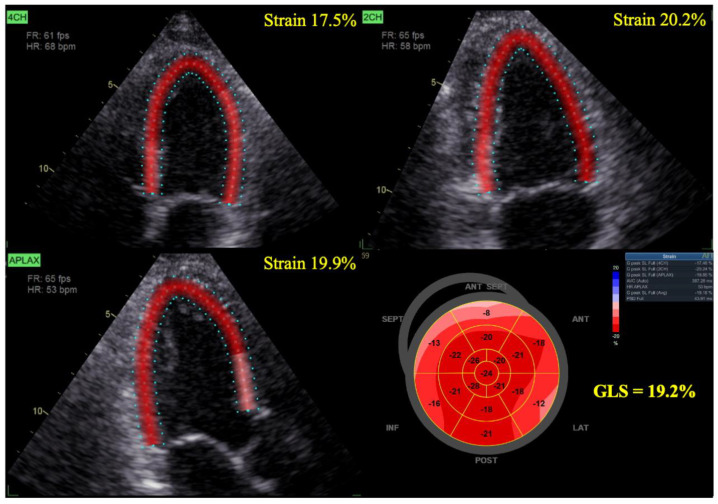
Measurement of GLS. Measurement of GLS by speckle−tracking echocardiography, where the regional strain map is superimposed on grayscale two−dimensional echocardiographic images in three apical long−axis views. GLS, global longitudinal strain.

GLS is an average of the LS of all myocardial segments. As the myocardial muscle contracts (and shortens), myocardial strain is expressed as a negative value. Strain measurements are obtained from echocardiographic images taken in the apical long-axis four-chamber and two-chamber views. Moreover, GLS is measured automatically, making it highly reproducible and easy to measure (Figure 1).

In clinical studies, GLS is presented as an absolute value, and percentage changes in LS are calculated according to the following equation: ([strain value at follow-up—strain value at baseline] × 100 ÷ strain value at baseline). Generally, percentage variations (relative changes) in strain values reflect the absolute values of strain parameters, with negative and positive variations indicating worsening and improving deformations, respectively. A previous meta-analysis defined normal GLS as −18%, but its load dependence stipulates that values ranging from −18% to −16% can be considered normal [30]. However, GLS must be measured with the same machine and software because the values may vary among vendors.

GLS is widely used in the field of cardiology generally, where it has demonstrated value in detecting subtle myocardial dysfunction, diagnosing disease, predicting disease progression, and allowing for early pharmacological intervention. GLS has demonstrated its usefulness in a number of cardiac diseases, including cardiomyopathy, myocardial infarction, HF, hypertension, and acute coronary syndrome [31,32,33,34]. Correlations between GLS and deleterious myocardial changes, prognostic predictors, and cardiovascular outcomes have been established in various diseases [31,32,33,34,35,36]. Therefore, it is not surprising that GLS assessment is recommended in the current cardio-oncology guidelines of the European Society of Cardiology [18]. In these guidelines, baseline transthoracic echocardiography is recommended for all patients with cancer who are at high risk or very high risk of cardiovascular toxicity before starting cancer therapy (Class I, Level of Evidence C), and GLS measurement is recommended for all patients who undergo echocardiography (Class I, Level of Evidence C). The definitions of asymptomatic and symptomatic CTRCD are shown in Table 1.

**Table 1:** Inclusion of GLS in the European Society of Cardiology’s definition of CTRCD.

*CTRCD*		
***Symptomatic CTRCD (HF) ^a^***	Very severe	HF requiring inotropic support, mechanical circulatory support, or transplantation
	Severe	HF hospitalization
	Moderate	Requirement for outpatient intensification of diuretics and HF treatment
	Mild	Mild HF symptoms; no intensification of therapy required
***Asymptomatic CTRCD***	Severe	New reduction in LVEF to <40%
	Moderate	New reduction in LVEF of ≥10% to 40–49% or new reduction in LVEF of <10% to an LVEF of 40–49%, and either new relative decline in GLS of >15% from baseline or new increase in cTnI/cTnT, BNP, or NT-proBNP
	Mild	LVEF of ≥50% and new relative decline in GLS of >15% from baseline and/or new increase in cTnI/cTnT, BNP, or NT-proBNP

Adapted from the 2022 European Society of Cardiology guidelines on cardio-oncology (Lyon et al., 2022) [18]. ^a^ With LVEF and supportive diagnostic biomarkers based on the 2021 European Society of Cardiology guidelines for the diagnosis and treatment of acute and chronic HF [37]. BNP, brain natriuretic peptide; cTnI, cardiac troponin I; cTnT, cardiac troponin T; CTRCD, cancer therapy-related cardiovascular dysfunction; GLS, global longitudinal strain; HF, heart failure; LVEF, left ventricular ejection fraction; NT-proBNP, N-terminal pro-brain natriuretic peptide.

The benefits of GLS assessment have been demonstrated in patients treated with anthracyclines and human epidermal growth factor receptor 2 (HER2)-targeted therapies, ICIs, radiation therapy, bevacizumab, carfilzomib, and hematopoietic stem cell transplantation in cardio-oncology [5,27,38,39,40,41,42,43,44,45,46]. It has been reported that basal LS decreases earlier than GLS in CTRCD in patients treated with anthracyclines [47]. Regional LS and GLS may be used as key measurement indices. In another study of lymphoblastic leukemia survivors, left ventricular systolic dysfunction was more easily detected by assessing GLS than by assessing LVEF [48]. In this study, 90 lymphoblastic leukemia survivors with a median time from diagnosis of 18 years were included. The prevalence of left ventricular systolic dysfunction was higher when using LS than when using LVEF. In another study, GLS was used to demonstrate that proton radiation therapy was superior to conventional radiation therapy in that it did not affect left ventricular function and was not associated with an increase in cardiac biomarkers [49]. In that study, 70 women treated with proton radiation therapy for breast cancer underwent GLS measurement before, 4 weeks after, and 2 months after the completion of proton radiation therapy. The SUCCOUR study evaluated whether there was a difference in the change in LVEF at 3 years between the group with LVEF-guided cardioprotective drug administration and the group with GLS-guided cardioprotective drug administration after anthracycline treatment [50]. This study was the first randomized controlled trial to compare GLS with LVEF in patients treated with cardiotoxic chemotherapy agents. The difference in LVEF from baseline to 3 years was not significantly different between the two groups. This finding suggests that risk stratification is important because of the low frequency of CTRCD. At 1 year, the proportion of patients with CTRCD was lower in the GLS-guided group, reflecting the importance of identifying subclinical left ventricular dysfunction and initiating treatment [29].

Although GLS is a robust and sensitive marker of CTRCD, image quality limitations can arise when GLS is measured by echocardiography [51]. In a previous study, 77 patients with breast cancer underwent echocardiography at 3-month intervals for 1 year before and after the initiation of chemotherapy. Image quality was classified as optimal, suboptimal, or inadequate. Of the 376 examinations performed, image quality was optimal in only 52%, suboptimal in 42%, and inadequate in 6%. Interobserver reproducibility in the optimal group was 0.91, while in the suboptimal group it was 0.21. This is important because changes in GLS may be missed if the image quality is suboptimal. Despite the limitations of echocardiographic measurement being recognized, Russo et al. [52] and Mirza et al. [53] concluded that the performance of echocardiography and cardiac magnetic resonance imaging (MRI) are comparable.

## 4. ICIs and Cardiovascular Complications

ICIs inhibit T-cell (programmed cell death protein 1 [PD-1] or cytotoxic T lymphocyte abtugen-4 [CTLA-4]) and tumor cell (programmed cell death-ligand 1 [PD-L1]) receptors, allowing T-cells to attack cancer cells. Although ICIs are treated as monotherapy, there are also combination therapies of ICIs that further improve clinical outcomes. They are also used in combination with other cancer drug therapies. ICIs are increasingly being used to treat a large number of cancer types [6,7]. However, ICIs attenuate self-tolerance to autoimmune-induced cells, resulting in systemic irAEs. These irAEs often affect various organs such as the colon, lungs, liver, thyroid, skin, and pituitary. Cardiovascular complications caused by ICIs are not common, but they may require the discontinuation or interruption of ICI therapy. Myocarditis, pericardial disease, vasculitis, coronary artery disease related to atherosclerosis, Takotsubo cardiomyopathy, and arrhythmia have been reported as possible cardiovascular complications [10,54,55,56]. Of these, myocarditis has a particularly high mortality rate and thus requires special attention.

The mechanism of ICI-induced myocarditis is not yet known, but it is considered to be related to CD8-positive T-cells [57,58,59]. Myocarditis associated with ICIs occurs in approximately 0.6–1.14% of patients [6,60,61], with a reported mortality rate of 27–50% [8,9,10]. Less severe ICI-induced myocarditis is now being diagnosed, and the reported mortality is trending downward, but it is still higher than that of myocarditis not associated with ICIs [62,63]. It has also been reported that patients who received higher doses of steroids early after diagnosis had better prognoses [64]. In addition to these factors, the prediction and early detection of ICI-induced myocarditis are important in situations where no preventive measures for ICI-induced myocarditis were taken. At present, ICI combination therapy is the most recognized risk factor for ICI-induced myocarditis, but no other definitive risk factors have been identified [10,38]. The onset of myocarditis often occurs early on in the course of treatment with ICIs, and numerous case reports have reported a median onset time of 27–57 days after the initiation of ICI therapy [10,40,65]. ICI-induced myocarditis is not common in the later remote phases after the initiation of ICI therapy [66,67]. Therefore, risk factors and early predictors of myocarditis should be identified.

Pericardial diseases include pericarditis, pericardial effusion, and cardiac tamponade. In a previous report [10], pericardial disease was most common in patients with lung cancer and was associated with radiation therapy and pericardial metastases. The vasculitides most commonly associated with ICIs were temporal arteritis and polymyalgia rheumatica, which were reported to be more common in older patients [10]. The median onset of vasculitis was 55 days after the start of ICI therapy. An association between coronary atherosclerosis and ICIs has also been reported [55,68,69]. In a patient group matched by age, prior cardiovascular events, and cancer type, a 3.3-fold increase in atherosclerotic cardiovascular events (myocardial infarction, coronary revascularization, ischemic stroke) was reported [55]. Arrhythmias have also been reported as cardiovascular complications associated with ICI therapy [10,54,55]. Arrhythmias that have been reported during ICI therapy include atrioventricular block, premature ventricular contraction, ventricular tachycardia, ventricular fibrillation, atrial fibrillation, and cardiac arrest, many of which may be related to myocarditis [70,71,72].

## 5. LS and ICIs

As a result of many published studies, the 2022 European Society of Cardiology guidelines recommend the following regarding GLS: GLS should be measured in all patients with cancer if available; GLS should be used to define mild or moderate asymptomatic CTRCD; and cardioprotective agents should be introduced if GLS is significantly decreased in patients undergoing treatment with anthracyclines or HER2-targeted therapies. Unfortunately, as with all drugs except anthracyclines and HER2 inhibitors, there are no comments specific to GLS measurement in patients treated with ICIs. However, recent studies have demonstrated that GLS is useful for the assessment of cardiac dysfunction in patients undergoing ICI therapy (Table 2).

**Figure 2:**
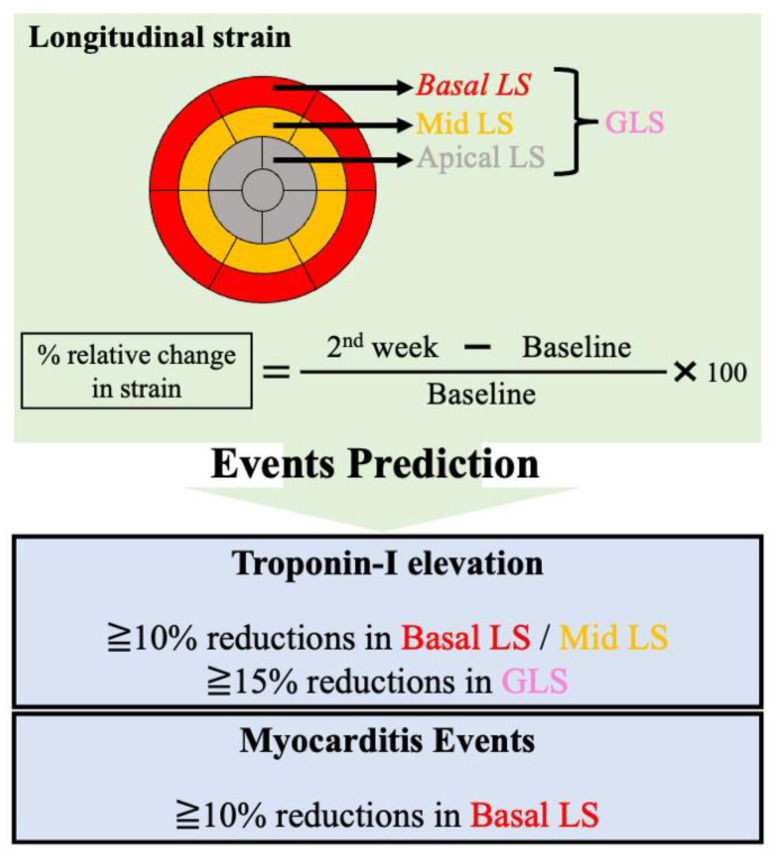
LS and cardiac events in patients undergoing ICI therapy. Summary of our study of patients treated with ICIs, in which a decrease of ≥10% in basal and mid-LS and of ≥15% in GLS were associated with troponin I elevation. In addition, two-thirds of patients with myocarditis demonstrated a decrease of ≥10% in basal LS. GLS, global longitudinal strain; ICI, immune checkpoint inhibitor; LS, longitudinal strain.

In a previous study, decreased GLS was observed in patients with ICI-induced myocarditis, and decreased GLS was strongly associated with major adverse cardiac events, regardless of LVEF [40]. Left ventricular dysfunction during ICI therapy is not necessarily due to myocarditis. GLS monitoring in patients with melanoma can detect subclinical left ventricular dysfunction induced by ICI therapy (in the absence of myocarditis) [76]. We previously reported that a decrease in GLS, basal LS, and mid-LS at week 2 was associated with cardiac troponin I elevation in patients undergoing ICI therapy [73]. In addition, two-thirds of patients with myocarditis had decreased basal LS. Monitoring basal LS and GLS may allow for early risk stratification of myocardial damage in patients undergoing ICI therapy (Figure 2). In this study, regional LS was calculated on the basis of values in the basal (six segments), mid- (six segments), and apical (five segments) layers.

Mirza et al. [53] compared echocardiography with cardiac MRI to examine regional and global changes in myocardial morphology and function induced by ICIs. Eight patients treated with ICIs underwent echocardiography and cardiac MRI to measure myocardial strain and strain rates, and the obtained measurements were compared between the two techniques. Echocardiography revealed a reduction in mean systolic LS in patients treated with ICIs. LS scores were comparable between echocardiography and cardiac MRI, suggesting a good coincidence between these two imaging methods. Moreover, ICI-treated patients had significantly lower LS and strain rates at the basal and mid-layers compared with controls. Thus, GLS and regional LS may be useful as diagnostic and predictive indices for ICI-induced myocarditis. Therefore, further studies examining the relationship between LS and ICI-induced myocarditis are expected in the future. In another study, cardiac MRI was performed at baseline and 3 months after the initiation of ICI therapy. The results also showed a decrease in LS in patients undergoing ICI therapy [74]. According to Mirza et al., echocardiography and cardiac MRI demonstrate comparable performance in patients undergoing ICI therapy [53]. Therefore, although some studies have evaluated LS by MRI, the evaluation of LS by echocardiography is cheaper, faster, and easier. As the measurement of LS by echocardiography is easy in real-world clinical practice, utilizing LS may be a good option for patients undergoing ICI therapy.

## 6. Conclusions

In conclusion, CTRCD is an important cause of morbidity in patients undergoing cancer therapy, and its incidence is likely to increase as patient survival is extended, the incidence of chronic underlying comorbidities increases, and novel cancer therapies are continuously developed. GLS is indicated as a useful echocardiographic measure that can detect subclinical CTRCD, and it is recommended by the European Society of Cardiology guidelines. GLS appears to be more sensitive to the detection of subclinical cardiac dysfunction than LVEF. Echocardiography and cardiac MRI demonstrate comparable performance in measuring GLS, although interobserver variation has been reported with echocardiography. Early diagnosis or prediction of irAEs associated with ICIs assessed by measuring LS may enable the safe use of ICIs in patients with cancer. In the future, LS may play a role as one of the key clinical factors in multifactorial analysis including multiomics and deep phenotyping approaches (network medicine strategy) for risk stratification of cardiac damages in patients with cancer therapy [77].

## Figures and Tables

**Table 2:** Summary of studies evaluating the relationship between LS and ICI therapy.

Publication	Imaging Modality (Timing)	Number of Patients	Details of ICIs	Key Message
Awadalla et al. 2020 [40]	Echocardiography (at baseline and upon diagnosis of myocarditis)	Patients with ICI-induced myocarditis (*n* = 101) and patients treated with ICIs (*n* = 92).	Patients with ICI-induced myocarditis Anti-PD1 (*n* = 78), anti-CTLA4 (*n* = 11), anti-PDL1 (*n* = 12), combination therapy (*n* = 27) Patients treated with ICIs Anti-PD1 (*n* = 73), anti-CTLA4 (*n* = 17), anti-PDL1 (*n* = 4), combination therapy (*n* = 6)	More than half of the patients with ICI-induced myocarditis had preserved LVEF, but GLS was decreased after ICI administration in the group of patients with ICI-induced myocarditis compared with baseline. In patients with ICI-induced myocarditis, GLS was associated with major cardiac events.
Mirza et al. 2022 [53]	Cardiac MRI and echocardiography (after initiation of ICIs, 24–48 h apart)	Patients with ICI cardiotoxicity (*n* = 8) and control subjects (*n* = 16).	Pembrolizumab (*n* = 3), nivolumab (*n* = 2), nivolumab and ipilimab (*n* = 1), avelumab (*n* = 1), duralumab (*n* = 1), ipilimab (*n* = 1)	Global, basal, and mid-LS were lower in patients treated with ICIs compared with controls. When comparing cardiac MRI with echocardiography, there was a strong coincidence between the two modalities in detecting changes in myocardial contractility and relaxation.
Tamura et al. 2022 [73]	Echocardiography (at baseline and 2 weeks after initiation of ICIs)	Patients treated with ICIs (*n* = 129). Of those, patients with troponin I elevation (*n* = 18) and ICI-associated myocarditis (*n* = 6).	Nivolumab (*n* = 58), pembrolizumab (*n* = 53), atezolizumab (*n* = 13), duralumab (*n* = 3), nivolumab and ipilimab (*n* = 2)	Early relative decline of >10% in basal and mid-LS and of >15% in GLS was associated with increased cardiac troponin I. Two thirds of patients with ICI-induced myocarditis had an early relative decline of ≥10% in basal LS.
Faron et al. 2021 [74]	Cardiac MRI (at baseline and 3 months after initiation of ICIs)	Patients treated with ICIs (*n* = 22).	Pembrolizumab (*n* = 9), nivolumab (*n* = 6), nivolumab and ipilimab (*n* = 4), Cemiplimab (*n* = 2), duralumab (*n* = 1)	There was an increase in markers of myocardial edema on follow-up cardiac MRI compared with baseline cardiac MRI. There was a significant decrease in LS on follow-up cardiac MRI.
Higgins et al. 2021 [75]	Cardiac MRI (at diagnosis of ICI cardiotoxicity)	Patients with ICI cardiotoxicity (*n* = 20).	pembrolizumab (*n* = 6), nivolumab and ipilimab (*n* = 5), nivolumab (*n* = 4), duralumab (*n* = 2), tremelimumab (*n* = 2), atezolizumab (*n* = 1), ipilimab (*n* = 1)	Cardiac MRI showed a moderate correlation between GLS and left ventricular end-systolic volume index, cardiac index, and LVEF.

CTLA4, cytotoxic T-lymphocyte-associated protein 4; GLS, global longitudinal strain; ICI, immune checkpoint inhibitor; LS, longitudinal strain; LVEF, left ventricular ejection fraction; MRI, magnetic resonance imaging; PD1, programmed cell death protein 1; PDL1, programmed death-ligand 1.

## Data Availability

Data sharing is not applicable.

## References

[1] Hurtado-de-Mendoza D., Loaiza-Bonilla A., Bonilla-Reyes P.A., Tinoco G., Alcorta R. (2017). Cardio-oncology: Cancer therapy-related cardiovascular complications in a molecular targeted era: New concepts and perspectives. Cureus.

[2] Mavrogeni S.I., Sfendouraki E., Markousis-Mavrogenis G., Rigopoulos A., Noutsias M., Kolovou G., Angeli C., Tousoulis D. (2019). Cardio-oncology, the myth of Sisyphus, and cardiovascular disease in breast cancer survivors. Heart Fail. Rev..

[3] Palmieri V., Vietri M.T., Montalto A., Montisci A., Donatelli F., Coscioni E., Napoli C. (2023). Cardiotoxicity, Cardioprotection, and Prognosis in Survivors of Anticancer Treatment Undergoing Cardiac Surgery: Unmet Needs. Cancers.

[4] Felker G.M., Thompson R.E., Hare J.M., Hruban R.H., Clemetson D.E., Howard D.L., Baughman K.L., Kasper E.K. (2000). Underlying causes and long-term survival in patients with initially unexplained cardiomyopathy. N. Engl. J. Med..

[5] Curigliano G., Lenihan D., Fradley M., Ganatra S., Barac A., Blaes A., Herrmann J., Porter C., Lyon A.R., Lancellotti P. (2020). Management of cardiac disease in cancer patients throughout oncological treatment: ESMO consensus recommendations. Ann. Oncol..

[6] Mahmood S.S., Fradley M.G., Cohen J.V., Nohria A., Reynolds K.L., Heinzerling L.M., Sullivan R.J., Damrongwatanasuk R., Chen C.L., Gupta D. (2018). Myocarditis in patients treated with immune checkpoint inhibitors. J. Am. Coll. Cardiol..

[7] Haslam A., Prasad V. (2019). Estimation of the percentage of US patients with cancer who are eligible for and respond to checkpoint inhibitor immunotherapy drugs. JAMA Netw. Open.

[8] Zlotoff D.A., Hassan M.Z.O., Zafar A., Alvi R.M., Awadalla M., Mahmood S.S., Zhang L., Chen C.L., Ederhy S., Barac A. (2021). Electrocardiographic features of immune checkpoint inhibitor associated myocarditis. J. Immunother. Cancer.

[9] Brumbaugh A.D., Narurkar R., Parikh K., Fanucchi M., Frishman W.H. (2019). Cardiac immune-related adverse events in immune checkpoint inhibition therapy. Cardiol. Rev..

[10] Salem J.E., Manouchehri A., Moey M., Lebrun-Vignes B., Bastarache L., Pariente A., Gobert A., Spano J.P., Balko J.M., Bonaca M.P. (2018). Cardiovascular toxicities associated with immune checkpoint inhibitors: An observational, retrospective, pharmacovigilance study. Lancet Oncol..

[11] Cardinale D., Iacopo F., Cipolla C.M. (2020). Cardiotoxicity of anthracyclines. Front. Cardiovasc. Med..

[12] Menna P., Salvatorelli E. (2017). Primary prevention strategies for anthracycline cardiotoxicity: A brief overview. Chemotherapy.

[13] Mercurio V., Pirozzi F., Lazzarini E., Marone G., Rizzo P., Agnetti G., Tocchetti C.G., Ghigo A., Ameri P. (2016). Models of heart failure based on the cardiotoxicity of anticancer drugs. J. Card. Fail..

[14] Minotti G., Menna P., Salvatorelli E., Cairo G., Gianni L. (2004). Anthracyclines: Molecular advances and pharmacologic developments in antitumor activity and cardiotoxicity. Pharmacol. Rev..

[15] Sawyer D.B. (2013). Anthracyclines and heart failure. N. Engl. J. Med..

[16] Cardinale D., Colombo A., Bacchiani G., Tedeschi I., Meroni C.A., Veglia F., Civelli M., Lamantia G., Colombo N., Curigliano G. (2015). Early detection of anthracycline cardiotoxicity and improvement with heart failure therapy. Circulation.

[17] Onishi T., Fukuda Y., Miyazaki S., Yamada H., Tanaka H., Sakamoto J., Daimon M., Izumi C., Nonaka A., Nakatani S. (2021). Practical guidance for echocardiography for cancer therapeutics-related cardiac dysfunction. J. Echocardiogr..

[18] Lyon A.R., López-Fernández T., Couch L.S., Asteggiano R., Aznar M.C., Bergler-Klein J., Boriani G., Cardinale D., Cordoba R., Cosyns B. (2022). 2022 ESC Guidelines on cardio-oncology developed in collaboration with the European Hematology Association (EHA), the European Society for Therapeutic Radiology and Oncology (ESTRO) and the International Cardio-Oncology Society (IC-OS). Eur. Heart J..

[19] Tan-Chiu E., Yothers G., Romond E., Geyer C.E., Ewer M., Keefe D., Shannon R.P., Swain S.M., Brown A., Fehrenbacher L. (2005). Assessment of cardiac dysfunction in a randomized trial comparing doxorubicin and cyclophosphamide followed by paclitaxel, with or without trastuzumab as adjuvant therapy in node-positive, human epidermal growth factor receptor 2-overexpressing breast cancer: NSABP B-31. J. Clin. Oncol..

[20] Perez E.A., Suman V.J., Davidson N.E., Sledge G.W., Kaufman P.A., Hudis C.A., Martino S., Gralow J.R., Dakhil S.R., Ingle J.N. (2008). Cardiac safety analysis of doxorubicin and cyclophosphamide followed by paclitaxel with or without trastuzumab in the North Central Cancer Treatment Group N9831 adjuvant breast cancer trial. J. Clin. Oncol..

[21] Morelli M.B., Bongiovanni C., Da Pra S., Miano C., Sacchi F., Lauriola M., D’Uva G. (2022). Cardiotoxicity of anticancer drugs: Molecular mechanisms and strategies for cardioprotection. Front. Cardiovasc. Med..

[22] Vaduganathan M., Prasad V. (2016). Cardiovascular risk assessment in oncological clinical trials: Is there a role for centralized events adjudication?. Eur. J. Heart Fail..

[23] Marwick T.H. (2022). Global longitudinal strain monitoring to guide cardioprotective medications during anthracycline treatment. Curr. Oncol. Rep..

[24] Oikonomou E.K., Kokkinidis D.G., Kampaktsis P.N., Amir E.A., Marwick T.H., Gupta D., Thavendiranathan P. (2019). Assessment of prognostic value of left ventricular global longitudinal strain for early prediction of chemotherapy-induced cardiotoxicity: A systematic review and meta-analysis. JAMA Cardiol..

[25] Gripp E.A., Oliveira G.E., Feijó L.A., Garcia M.I., Xavier S.S., Sousa A.S. (2018). Global Longitudinal Strain Accuracy for Cardiotoxicity Prediction in a Cohort of Breast Cancer Patients during Anthracycline and/or Trastuzumab Treatment. Arq. Bras. Cardiol..

[26] Patel J., Rikhi R., Hussain M., Ayoub C., Klein A., Collier P., Moudgil R. (2020). Global longitudinal strain is a better metric than left ventricular ejection fraction: Lessons learned from cancer therapeutic-related cardiac dysfunction. Curr. Opin. Cardiol..

[27] Yamada K., Tamura Y., Taniguchi H., Furukawa A., Iwasawa J., Yada H., Kawamura A., Tamura Y. (2022). Usefulness of global longitudinal strain-guided management in preventing human epidermal growth factor receptor 2 (HER2) inhibitor-induced myocardial damage. Circ. Rep..

[28] Santoro C., Esposito R., Lembo M., Sorrentino R., De Santo I., Luciano F., Casciano O., Giuliano M., De Placido S., Trimarco B. (2019). Strain-oriented strategy for guiding cardioprotection initiation of breast cancer patients experiencing cardiac dysfunction. Eur. Heart J. Cardiovasc. Imaging.

[29] Thavendiranathan P., Negishi T., Somerset E., Negishi K., Penicka M., Lemieux J., Aakhus S., Miyazaki S., Shirazi M., Galderisi M. (2021). Strain-guided management of potentially cardiotoxic cancer therapy. J. Am. Coll. Cardiol..

[30] D’Elia N., Caselli S., Kosmala W., Lancellotti P., Morris D., Muraru D., Takeuchi M., van den Bosch A., van Grootel R.W.J., Villarraga H. (2020). Normal global longitudinal strain: An individual patient meta-analysis. JACC Cardiovasc. Imaging.

[31] Bière L., Donal E., Terrien G., Kervio G., Willoteaux S., Furber A., Prunier F. (2014). Longitudinal strain is a marker of microvascular obstruction and infarct size in patients with acute ST-segment elevation myocardial infarction. PLoS ONE.

[32] He J., Yang W., Wu W., Li S., Yin G., Zhuang B., Xu J., Sun X., Zhou D., Wei B. (2021). Early diastolic longitudinal strain rate at MRI and outcomes in heart failure with preserved ejection fraction. Radiology.

[33] Stoichescu-Hogea G., Buleu F.N., Christodorescu R., Sosdean R., Tudor A., Ember A., Brie D.M., Drăgan S. (2021). Contribution of global and regional longitudinal strain for clinical assessment of HFpEF in coronary and hypertensive patients. Medicina.

[34] Mawla T.S.A., Wanees W.S.A., Fattah E.M.A., El Khashab K.A., Momtaz O.M. (2023). Diagnostic accuracy of global longitudinal strain in prediction of severity and extent of coronary artery stenosis in patients with acute coronary syndrome. Acta Cardiol..

[35] Laufer-Perl M., Arnold J.H., Moshkovits Y., Havakuk O., Shmilovich H., Chausovsky G., Sivan A., Szekely Y., Arbel Y., Banai S. (2022). Evaluating the role of left ventricle global longitudinal strain in myocardial perfusion defect assessment. Int. J. Cardiovasc. Imaging.

[36] Sun W., Shen X., Wang J., Zhu S., Zhang Y., Wu C., Xie Y., Yang Y., Dong N., Wang G. (2021). Association between 2D- and 3D-speckle-tracking longitudinal strain and cardiovascular magnetic resonance evidence of diffuse myocardial fibrosis in heart transplant recipients. Front. Cardiovasc. Med..

[37] McDonagh T.A., Metra M., Adamo M., Gardner R.S., Baumbach A., Böhm M., Burri H., Butler J., Čelutkienė J., Chioncel O. (2021). 2021 ESC Guidelines for the diagnosis and treatment of acute and chronic heart failure. Eur. Heart J..

[38] Čelutkienė J., Pudil R., López-Fernández T., Grapsa J., Nihoyannopoulos P., Bergler-Klein J., Cohen-Solal A., Farmakis D., Tocchetti C.G., von Haehling S. (2020). Role of cardiovascular imaging in cancer patients receiving cardiotoxic therapies: A position statement on behalf of the Heart Failure Association (HFA), the European Association of Cardiovascular Imaging (EACVI) and the Cardio-Oncology Council of the European Society of Cardiology (ESC). Eur. J. Heart Fail..

[39] Pudil R., Mueller C., Čelutkienė J., Henriksen P.A., Lenihan D., Dent S., Barac A., Stanway S., Moslehi J., Suter T.M. (2020). Role of serum biomarkers in cancer patients receiving cardiotoxic cancer therapies: A position statement from the Cardio-Oncology Study Group of the Heart Failure Association and the Cardio-Oncology Council of the European Society of Cardiology. Eur. J. Heart Fail..

[40] Awadalla M., Mahmood S.S., Groarke J.D., Hassan M.Z.O., Nohria A., Rokicki A., Murphy S.P., Mercaldo N.D., Zhang L., Zlotoff D.A. (2020). Global longitudinal strain and cardiac events in patients with immune checkpoint inhibitor-related myocarditis. J. Am. Coll. Cardiol..

[41] Trivedi S.J., Choudhary P., Lo Q., Sritharan H.P., Iyer A., Batumalai V., Delaney G.P., Thomas L. (2019). Persistent reduction in global longitudinal strain in the longer term after radiation therapy in patients with breast cancer. Radiother. Oncol..

[42] Xu X., Wang D., Yin Y., Dai H., Chen L., Xue J., Li Q. (2023). Role of global longitudinal strain in evaluating radiotherapy-induced early cardiotoxicity in breast cancer: A meta-analysis. Kardiol. Pol..

[43] Lo Q., Hee L., Batumalai V., Allman C., MacDonald P., Delaney G.P., Lonergan D., Thomas L. (2015). Subclinical cardiac dysfunction detected by strain imaging during breast irradiation with persistent changes 6 weeks after treatment. Int. J. Radiat. Oncol. Biol. Phys..

[44] Sonaglioni A., Albini A., Fossile E., Pessi M.A., Nicolosi G.L., Lombardo M., Anzà C., Ambrosio G. (2020). Speckle-tracking echocardiography for cardioncological evaluation in bevacizumab-treated colorectal cancer patients. Cardiovasc. Toxicol..

[45] Mingrone G., Astarita A., Airale L., Maffei I., Cesareo M., Crea T., Bruno G., Leone D., Avenatti E., Catarinella C. (2021). Effects of carfilzomib therapy on left ventricular function in multiple myeloma patients. Front. Cardiovasc. Med..

[46] Oliveira G.H., Al-Kindi S.G., Guha A., Dey A.K., Rhea I.B., deLima M.J. (2021). Cardiovascular risk assessment and management of patients undergoing hematopoietic cell transplantation. Bone Marrow Transplant..

[47] Saijo Y., Kusunose K., Okushi Y., Yamada H., Toba H., Sata M. (2020). Relationship between regional left ventricular dysfunction and cancer-therapy-related cardiac dysfunction. Heart.

[48] Gonzalez-Manzanares R., Castillo J.C., Molina J.R., Ruiz-Ortiz M., Mesa D., Ojeda S., Anguita M., Pan M. (2022). Automated global longitudinal strain assessment in long-term survivors of childhood acute lymphoblastic leukemia. Cancers.

[49] Hassan M.Z.O., Awadalla M., Tan T.C., Scherrer-Crosbie M., Bakar R.B., Drobni Z.D., Zarif A., Gilman H.K., Supraja S., Nikolaidou S. (2023). Serial measurement of global longitudinal strain among women with breast cancer treated with proton radiation therapy: A prospective trial for 70 patients. Int. J. Radiat. Oncol. Biol. Phys..

[50] Negishi T., Thavendiranathan P., Penicka M., Lemieux J., Murbraech K., Miyazaki S., Shirazi M., Santoro C., Cho G.Y., Popescu B.A. (2023). Cardioprotection using strain-guided management of potentially cardiotoxic cancer therapy: 3-year results of the SUCCOUR trial. JACC Cardiovasc. Imaging.

[51] Iida N., Tajiri K., Ishizu T., Sasamura-Koshizuka R., Nakajima H., Kawamatsu N., Sato K., Yamamoto M., Machino-Ohtsuka T., Bando H. (2022). Echocardiography image quality of global longitudinal strain in cardio-oncology: A prospective real-world investigation. J. Echocardiogr..

[52] Russo I., Micotti E., Fumagalli F., Magnoli M., Ristagno G., Latini R., Staszewsky L. (2019). A novel echocardiographic method closely agrees with cardiac magnetic resonance in the assessment of left ventricular function in infarcted mice. Sci. Rep..

[53] Mirza J., Sunder S.S., Karthikeyan B., Kattel S., Pokharel S., Quigley B., Sharma U.C. (2022). Echocardiographic and cardiac MRI comparison of longitudinal strain and strain rate in cancer patients treated with immune checkpoint inhibitors. J. Pers. Med..

[54] D’Souza M., Nielsen D., Svane I.M., Iversen K., Rasmussen P.V., Madelaire C., Fosbøl E., Køber L., Gustafsson F., Andersson C. (2021). The risk of cardiac events in patients receiving immune checkpoint inhibitors: A nationwide Danish study. Eur. Heart J..

[55] Escudier M., Cautela J., Malissen N., Ancedy Y., Orabona M., Pinto J., Monestier S., Grob J.J., Scemama U., Jacquier A. (2017). Clinical features, management, and outcomes of immune checkpoint inhibitor-related cardiotoxicity. Circulation.

[56] Drobni Z.D., Alvi R.M., Taron J., Zafar A., Murphy S.P., Rambarat P.K., Mosarla R.C., Lee C., Zlotoff D.A., Raghu V.K. (2020). Association between immune checkpoint inhibitors with cardiovascular events and atherosclerotic plaque. Circulation.

[57] Axelrod M.L., Meijers W.C., Screever E.M., Qin J., Carroll M.G., Sun X., Tannous E., Zhang Y., Sugiura A., Taylor B.C. (2022). T cells specific for α-myosin drive immunotherapy-related myocarditis. Nature.

[58] Palaskas N.L., Segura A., Lelenwa L., Siddiqui B.A., Subudhi S.K., Lopez-Mattei J., Durand J.B., Deswal A., Zhao B., Maximilian Buja L. (2021). Immune checkpoint inhibitor myocarditis: Elucidating the spectrum of disease through endomyocardial biopsy. Eur. J. Heart Fail..

[59] Zhu H., Galdos F.X., Lee D., Waliany S., Huang Y.V., Ryan J., Dang K., Neal J.W., Wakelee H.A., Reddy S.A. (2022). Identification of pathogenic immune cell subsets associated with checkpoint inhibitor-induced myocarditis. Circulation.

[60] Johnson D.B., Balko J.M., Compton M.L., Chalkias S., Gorham J., Xu Y., Hicks M., Puzanov I., Alexander M.R., Bloomer T.L. (2016). Fulminant myocarditis with combination immune checkpoint blockade. N. Engl. J. Med..

[61] Agostinetto E., Eiger D., Lambertini M., Ceppi M., Bruzzone M., Pondé N., Plummer C., Awada A.H., Santoro A., Piccart-Gebhart M. (2021). Cardiotoxicity of immune checkpoint inhibitors: A systematic review and meta-analysis of randomised clinical trials. Eur. J. Cancer.

[62] Grün S., Schumm J., Greulich S., Wagner A., Schneider S., Bruder O., Kispert E.M., Hill S., Ong P., Klingel K. (2012). Long-term follow-up of biopsy-proven viral myocarditis: Predictors of mortality and incomplete recovery. J. Am. Coll. Cardiol..

[63] Gräni C., Eichhorn C., Bière L., Murthy V.L., Agarwal V., Kaneko K., Cuddy S., Aghayev A., Steigner M., Blankstein R. (2017). Prognostic value of cardiac magnetic resonance tissue characterization in risk stratifying patients with suspected myocarditis. J. Am. Coll. Cardiol..

[64] Zhang L., Zlotoff D.A., Awadalla M., Mahmood S.S., Nohria A., Hassan M.Z.O., Thuny F., Zubiri L., Chen C.L., Sullivan R.J. (2020). Major adverse cardiovascular events and the timing and dose of corticosteroids in immune checkpoint inhibitor-associated myocarditis. Circulation.

[65] Moslehi J.J., Salem J.E., Sosman J.A., Lebrun-Vignes B., Johnson D.B. (2018). Increased reporting of fatal immune checkpoint inhibitor-associated myocarditis. Lancet.

[66] Nguyen A.T., Berry G.J., Witteles R.M., Le D.T., Wu S.M., Fisher G.A., Zhu H. (2022). Late-onset immunotherapy-induced myocarditis 2 years after checkpoint inhibitor initiation. JACC Cardio Oncol..

[67] Tamura Y., Tamura Y., Yamada K., Taniguchi H., Iwasawa J., Yada H., Kawamura A. (2022). Routine assessment of cardiotoxicity in patients undergoing long-term immune checkpoint inhibitor therapy. Heart Vessel..

[68] Chitturi K.R., Xu J., Araujo-Gutierrez R., Bhimaraj A., Guha A., Hussain I., Kassi M., Bernicker E.H., Trachtenberg B.H. (2019). Immune checkpoint inhibitor-related adverse cardiovascular events in patients with lung cancer. JACC Cardio Oncol..

[69] Cautela J., Rouby F., Salem J.E., Alexandre J., Scemama U., Dolladille C., Cohen A., Paganelli F., Ederhy S., Thuny F. (2020). Acute coronary syndrome with immune checkpoint inhibitors: A proof-of-concept case and pharmacovigilance analysis of a life-threatening adverse event. Can. J. Cardiol..

[70] Moslehi J.J., Johnson D.B., Sosman J.A. (2017). Myocarditis with immune checkpoint blockade. N. Engl. J. Med..

[71] Wang D.Y., Okoye G.D., Neilan T.G., Johnson D.B., Moslehi J.J. (2017). Cardiovascular toxicities associated with cancer immunotherapies. Curr. Cardiol. Rep..

[72] Rhea I.B., Oliveira G.H. (2018). Cardiotoxicity of novel targeted chemotherapeutic agents. Curr. Treat. Options Cardiovasc. Med..

[73] Tamura Y., Tamura Y., Takemura R., Yamada K., Taniguchi H., Iwasawa J., Yada H., Kawamura A. (2022). Longitudinal strain and troponin I elevation in patients undergoing immune checkpoint inhibitor therapy. JACC Cardio Oncol..

[74] Faron A., Isaak A., Mesropyan N., Reinert M., Schwab K., Sirokay J., Sprinkart A.M., Bauernfeind F.G., Dabir D., Pieper C.C. (2021). Cardiac MRI depicts immune checkpoint inhibitor-induced myocarditis: A prospective study. Radiology.

[75] Higgins A.Y., Arbune A., Soufer A., Ragheb E., Kwan J.M., Lamy J., Henry M., Cuomo J.R., Charifa A., Gallegos C. (2021). Left ventricular myocardial strain and tissue characterization by cardiac magnetic resonance imaging in immune checkpoint inhibitor associated cardiotoxicity. PLoS ONE.

[76] Mincu R.I., Pohl J., Mrotzek S., Michel L., Hinrichs L., Lampe L., Rassaf T., Totzeck M. (2020). Left ventricular global longitudinal strain reduction in patients with melanoma and extra-cardiac immune-related adverse events during immune checkpoint inhibitor therapy. Eur. Heart J..

[77] Benincasa G., Marfella R., Della Mura N., Schiano C., Napoli C. (2020). Strengths and Opportunities of Network Medicine in Cardiovascular Diseases. Circ. J..

